# A histological and micro-CT investigation in to the effect of NGF and EGF on the periodontal, alveolar bone, root and pulpal healing of replanted molars in a rat model - a pilot study

**DOI:** 10.1186/2196-1042-15-2

**Published:** 2014-01-06

**Authors:** Francesco Furfaro, Estabelle SM Ang, Ricky R Lareu, Kevin Murray, Mithran Goonewardene

**Affiliations:** 1 Department of Orthodontics, The University of Western Australia, Crawley, Western Australia 6009, Australia; 2 Department of Dentistry, The University of Western Australia, Crawley, Western Australia 6009, Australia; 3 Department of Medicine and Pharmacology, The University of Western Australia, Crawley, Western Australia 6009, Australia; 4 Department of Pharmacy, Curtin University, Bentley, Western Australia 6102, Australia; 5 Department of Mathematics and Statistics, The University of Western Australia, Crawley, Western Australia 6009, Australia

**Keywords:** Dental autotransplantation, Periodontal healing, Root healing, Pulpal regeneration, Nerve growth factor (NGF), Epidermal growth factor (EGF)

## Abstract

**Background:**

This study aims to investigate, utilising micro-computed tomography (micro-CT) and histology, whether the topical application of nerve growth factor (NGF) and/or epidermal growth factor (EGF) can enhance periodontal, alveolar bone, root and pulpal tissue regeneration while minimising the risk of pulpal necrosis, root resorption and ankylosis of replanted molars in a rat model.

**Methods:**

Twelve four-week-old male Sprague-Dawley rats were divided into four groups: sham, collagen, EGF and NGF. The maxillary right first molar was elevated and replanted with or without a collagen membrane impregnated with either the growth factors EGF or NGF, or a saline solution. Four weeks after replantation, the animals were sacrificed and the posterior maxilla was assessed using histological and micro-CT analysis. The maxillary left first molar served as the control for the corresponding right first molar.

**Results:**

Micro-CT analysis revealed a tendency for all replanted molars to have reduced root length, root volume, alveolar bone height and inter-radicular alveolar bone volume. It appears that the use of the collagen membrane had a negative effect while no positive effect was noted with the incorporation of EGF or NGF. Histologically, the incorporation of the collagen membrane was found to negatively affect pulpal, root, periodontal and alveolar bone healing with pulpal inflammation and hard tissue formation, extensive root resorption and alveolar bone fragmentation. The incorporation of EGF and NGF did not improve root, periodontal or alveolar bone healing. However, EGF was found to improve pulp vascularisation while NGF-improved pulpal architecture and cell organisation, although not to the level of the control group.

**Conclusions:**

Results indicate a possible benefit on pulpal vascularisation and pulpal cell organisation following the incorporation of EGF and NGF, respectively, into the alveolar socket of replanted molars in the rat model. No potential benefit of EGF and NGF was detected in periodontal or root healing, while the use of a collagen membrane carrier was found to have a negative effect on the healing response.

## Background

Tooth autotransplantation is a reliable treatment alternative for missing or damaged teeth [1]. Unlike the traditional prosthodontic options, a transplanted tooth preserves the dentoalveolar ridge, induces the formation of new supporting structures, continues root formation and erupts and maintains occlusal contacts with the opposing teeth while adapting to orofacial growth and development [2-4].

Factors exist, however, that limit the routine use of this technique. These include pulpal necrosis and inflammation, reduced root formation, root resorption and ankylosis [5,6]. The means of predictably reducing the complications associated with dental autotransplantation while extending the indications and timing boundaries dictated by the biological healing mechanisms of the pulp, root and periodontal tissues are required.

Past approaches included the use of periodontal ligament (PDL) stimulation techniques [7], connective tissue transplants [8], membrane barriers [9] and enamel matrix derivative (EMD) proteins [10,11]. More recent approaches involve the incorporation of naturally occurring growth factors into the dental transplant site with the aim of enhancing periodontal healing, root formation and pulpal regeneration. However, the limited number of studies performed to date utilising growth factors during dental autotransplantation have reported contrasting results.

Results obtained by Komatsu and co-workers suggest that the topical application of platelet-derived growth factor (PDGF) to replanted first molar teeth in the rat effectively promotes restoration of the support function of the healing PDL while minimising the risk of ankylosis [12]. Springer and colleagues found that the incorporation of bone morphogenetic protein-7 (BMP-7) into the tooth socket prior to replantation in minipigs improved the survival rate, but only when the PDL was partially traumatised. When there was minimal or total destruction of the PDL prior to replantation, there was no difference in survival rates amongst the control and BMP-7 groups [13]. In a series of studies performed by Sorensen et al. [14] and Wikesjo et al. [15] the topical application of bone morphogenetic protein-12 (BMP-12) to replanted teeth in Labrador mongrel dogs did not have an apparent effect on new cementum and PDL formation or on the presence and extent of ankylosis when compared to controls.

To date, no studies have looked into the effect of nerve growth factor (NGF) or epidermal growth factor (EGF) on the healing of pulpal, root, alveolar and periodontal tissues subsequent to tooth transplantation.

EGF enhances cellular proliferation and differentiation of epidermal and epithelial cells, fibroblasts, and cartilage and bone derived cells during growth, maturation and healing [16-19]. Following dentoalveolar trauma, it is speculated that circulating EGF is released from the platelets during blood clot formation where it mediates the recruitment of PDL precursor cells and their proliferation [20,21]. As the PDL precursor cells mature, the role of EGF changes to regulate the differentiation of hard-tissue forming cells and their synthetic activities [19].

NGF is a target-derived neurotrophic factor essential for the development, growth, survival, differentiation and maintenance of sympathetic and sensory neurones, including those of the dental pulp [22,23]. Emerging evidence indicates that NGF may have a broader physiological effect than regulating neuronal functions [24]. Studies demonstrate that NGF is involved in bone tissue healing by activating osteoblasts, tubular dentine formation by stimulating preodontoblasts and enhancing the proliferation and differentiation of PDL cells [25-28].

This study aims to investigate, utilising micro-computed tomography (micro-CT) and histology, whether the topical application of NGF and/or EGF can enhance periodontal, alveolar bone, root and pulpal tissue regeneration after autotransplantation while minimising the risk of pulpal necrosis, root resorption and ankylosis.

## Methods

### Animals

All experimental procedures administered to the animals were carried out in accordance with the protocol approved by the Animal Care and Veterinary Services Research committee of the University of Western Australia.

Twelve four-week-old male Sprague-Dawley rats were obtained from the Animal Resource Centre (Canningvale, WA, Australia). Animals were randomly assigned into the sham, collagen only, collagen-EGF and collagen-NGF treated groups. Each group consisted of three animals (Table 1), with the maxillary right first molar serving as the experimental side and the left molar serving as the untouched within sample control. Even though an untouched control would be ideal, animal ethics required animal numbers be minimised and the topical application would minimise any systemic factors.

**Table 1:** Summary of the experimental groups

**Group**	**Experimental plan**	**Number of animals**
Sham control	Molar replantation only - no collagen scaffold or growth factor	3
Collagen only	Molar replantation, collagen scaffold, no growth factor (saline)	3
EGF and collagen	Molar replantation, collagen scaffold impregnated with EGF	3
NGF and collagen	Molar replantation, collagen scaffold impregnated with NGF	3

All animals were housed at The University of Western Australia (QEII Medical Centre, M Block level 2) animal housing facility in accordance with the guidelines of the NHMRC Code of Practice for the care and use of animals for scientific purposes.

### Surgical procedure and tissue preparation

Prior to surgery, all animals were anaesthetised with an intra-peritoneal injection of ketamine hydrochloride (75 mg/kg) mixed with xylazine hydrochloride (10 mg/kg) diluted in sterile saline. To minimise postoperative pain, buprenorphine (0.05 mg/kg) and meloxicam (1 mg/kg) were administered shortly after the anaesthesia via subcutaneous injection.

**Figure 1:**
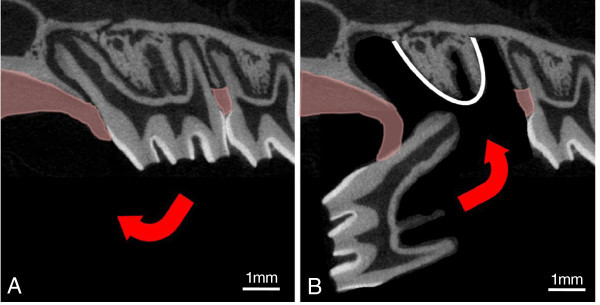
**Diagrammatic representation of the transplantation procedure.** Representation of the experimental replantation procedure adapted from Kvinnsland et al. [29]. **(A)** The right maxillary first molar is gently extracted and reflected mesially by 90° while the mesial attached gingiva is left intact. **(B)** It is then immediately replanted without (sham group) or with a collagen membrane placed at the base of the alveolar socket (white line) containing either saline, EGF or NGF.

All maxillary right first molars were elevated mesially at 90° by means of a custom-made dental elevator while maintaining the mesial gingival attachment in accordance with the protocol described by Kvinnsland and colleagues (Figure 1) [29]. The maxillary left first molar served as a control for the corresponding right first molar.

Depending on the experimental group (Table 1), a 2-mm-square piece of collagen membrane (Koken, Tokyo, Japan) soaked in sterile saline, NGF (R&D Systems, Minneapolis, MN, USA) at concentration of 0.5 mg/ml, or EGF (R&D Systems, Minneapolis, MN, USA) at concentration of 0.5 mg/ml was placed at the base of the socket prior to the replantation of the tooth (Figure 1B). Concentration used was based on a previous study demonstrating a therapeutic effect with EGF [30]. In the sham group, no collagen membrane was placed prior to replantation.

The rats received a soft diet for 1 week before being placed on the standard rat feed pellets for normal occlusal loading. Postoperative analgesics were administered by a subcutaneous injection of buprenorphine (0.05 mg/kg) and meloxicam (1 mg/kg) every 24 h for 3 days.

The animals were observed daily and their body weight were recorded 3 times a week to ensure normal growth and health. The animals were sacrificed 4 weeks after the surgery with methoxyflurane inhalation followed by cardiac injection of a lethal overdose of sodium pentobarbital (150 mg/mL). Four weeks was selected with previous studies which showed that the majority of periodontal and pulpal healing occurred within 2 to 4 weeks after transplantation [31,32]. A block resection of the maxilla was made and the specimens were fixed in 10% neutral buffered formalin (pH 7.4) overnight at 4°C.

### Micro-CT imaging and analysis

For three-dimensional analysis of the teeth and alveolar bone, fixed maxillary samples were scanned at the National Imaging Facility, Centre for Microscopy, Characterisation and Analysis (CMCA), The University of Western Australia, by means of a micro-CT system (SkyScan 1176 *in vivo* micro-CT, Kontich, Belgium). The specimens were scanned at a resolution of 9 μm, ensuring that all molar teeth and surrounding alveolar bone of the posterior maxilla were encompassed.

**Figure 2:**
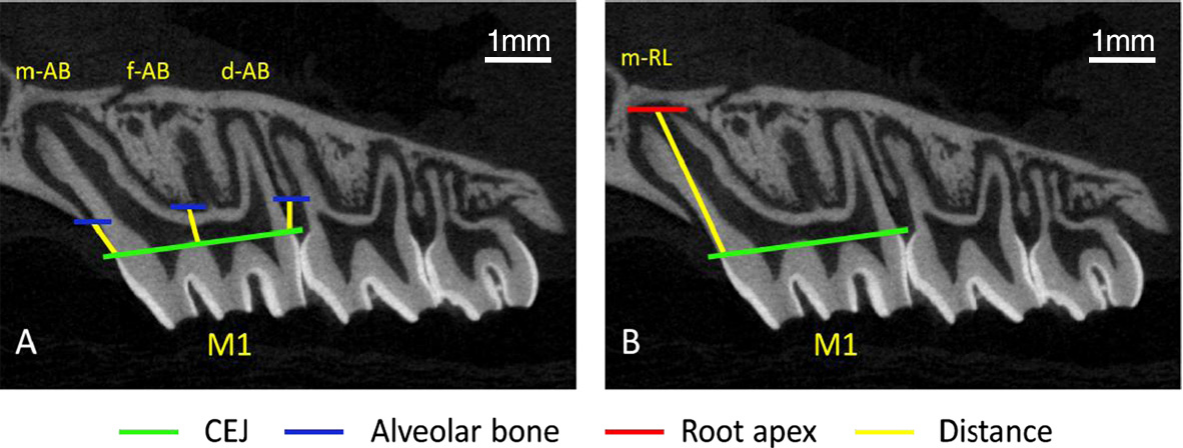
**Linear measurements to assess of alveolar bone height and root length from the micro-CT sections.** Linear measurements of alveolar bone loss (AB) and root length (RL) of the maxillary left and right first molars (M1). **(A)***Linear measurements* to assess alveolar bone loss were taken from the level of the cementoenamel junction (CEJ) to the alveolar bone crest mesial to the first molar (m-AB), at the furcation of the first molar distal to the mesial root (f-AB) and distal to the first molar (d-AB). **(B)** Measurement of the mesial root length (m-RL) from the CEJ to the mesial root apex was taken to assess the level of root development (Figure 2B). All teeth were analysed using the sagittal slice that contained the longest length of the mesial root. The mesial root only was assessed to eliminate trauma that may have been sustained to the distal roots during the luxation process.

**Figure 3:**
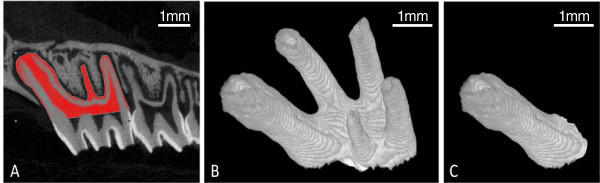
**Assessment of total root volume and mesial root volume. (A)** Root structure is carefully highlighted, ending at the CEJ, for every fifth sagittal slice containing the maxillary first molar roots. Intermediate slices were initially interpolated by morphing and then visually inspected, with the contours modified where necessary, to ensure that all root structure was included. **(B)** Reconstruction of the sagittal slices produced a 3-D representation of the entire root structure to allow assessment of root volume (RV). **(C)** The mesial root was then digitally resected from this rendering and its volume is analysed individually.

**Figure 4:**
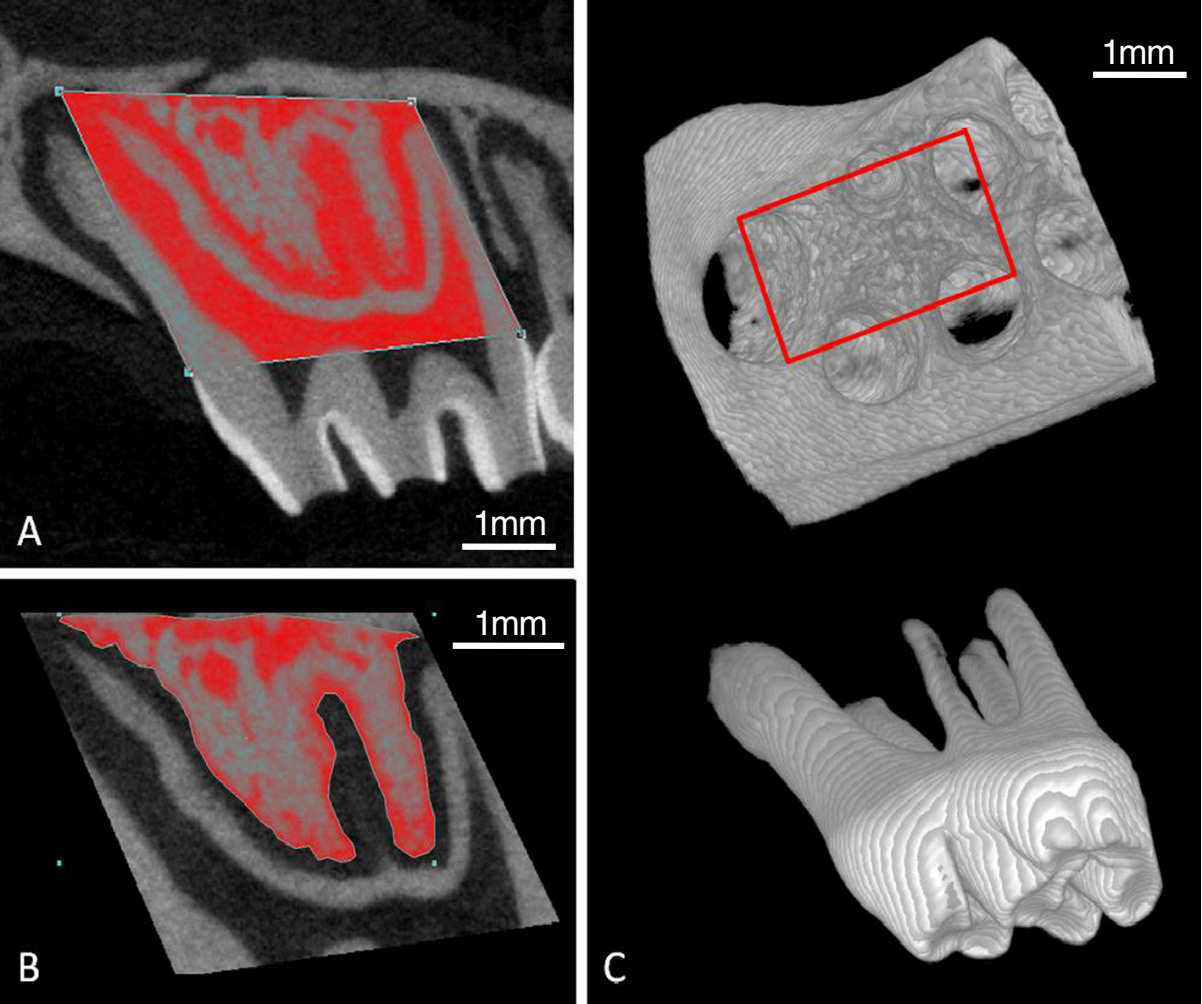
**Assessment of the inter-radicular alveolar bone volume of the maxillary right and left first molars. (A)** Volumetric assessment of the inter-radicular bone was determined by first drawing a region of interest (ROI) around the maxillary left first molar (control side) extending from the mesial and distal extension of the CEJ to the apex of the mesial and distobuccal roots. This is extended buccally and palatally to encompass the complete buccopalatal width of the mesial root. Due to the root and alveolar resorption observed in the maxillary right side (experimental side), the root apices of the right molar could not be used as reliable reference points to establish the ROI. Instead, the 3-dimensional ROI (3-D ROI) created for the control side is transferred to the right molar using the CEJ as a reproducible reference plane to produce an ROI that is identical for both the left and right molars of each animal. **(B)** Inter-radicular bone was carefully contoured for every fifth slice with the intermediate slices initially interpolated by morphing. Each slice was subsequently visually inspected and the contour was modified where deemed necessary, ensuring that all root structure, and any cortical both, were excluded. **(C)** The region of the resultant 3-D ROI (red rectangle) with the maxillary first molar removed and is used to determine tissue volume (TV), bone volume (BV) and bone volume fraction (BV/TV) of the inter-radicular alveolar bone.

The 3-D volume viewer and analyser software (CT-Analyser and CT-Volume, SkyScan, Kontich, Belgium) were used for the visualisation and quantification of 2-D and 3-D data on a personal computer output. Linear measurements to assess alveolar bone levels and volumetric measurements to assess root and inter-radicular bone were then performed using the protocols described in Figures 2, 3, 4, respectively.

### Histological preparation and analysis

After micro-CT imaging, the samples were washed in PBS and demineralised in 10% EDTA solution at pH 7.4 for 4 weeks. Before embedding in paraffin wax, the tissues were dehydrated through graded alcohol. Serial sagittal sections of 5 μm were made through the midline of the teeth allowing the mesial root, pulp chamber and inter-radicular alveolar bone to be observed simultaneously. The sections were stained with haematoxylin and eosin (H & E) prior to histological examination.

Descriptive analysis of the teeth focused on the continuation of root formation, the presence or absence of cementum covering the root surface, formation of PDL, presence or absence of root ankylosis and resorption, the quality of the bone surrounding the root and the vitality of the pulp cells.

### Statistical analysis

Formal statistical analyses were carried out using liner mixed models. A linear mixed model approach was taken in each instance with fixed factors group and side and their corresponding interaction. Random effect of individual within treatment was used. Estimated means for left and right percentage difference are provided by group, along with standard errors and *p* values for these comparisons. All analyses are carried out using R: a language and environment for statistical computing (2012).

## Results

### Micro-CT analysis

**Figure 5:**
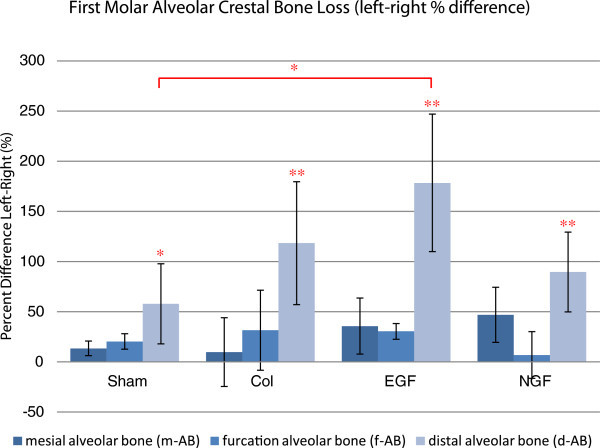
**Comparison of the left and right first molar alveolar bone levels.** Estimated mean percentage difference between the linear measurements of alveolar bone loss (m-AB, f-AB and d-AB) of the upper right and left first molars. Significant left-right differences were noted in the distal alveolar bone loss for all groups, while the only significant between-group difference occurred between sham and EGF. Error bars represent the standard error of the mean difference. *P < 0.05 and **P < 0.01.

*Linear measurements* of alveolar bone loss demonstrated differences between the untouched upper left and experimental upper right sides (Figure 5). These differences, however, are only significant for the measurement of the distal alveolar bone height (d-AB) with the experimental groups sham and EGF having a significant between-group difference.

**Figure 6:**
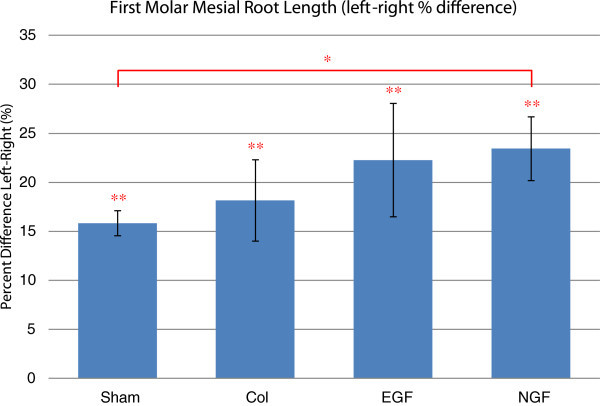
**Comparison of the left and right first molar mesial root length.** Estimated mean percentage difference between the length of the upper right and left first molar mesial roots. All experimental groups demonstrated a significant reduction in the final mesial root length of the upper right first molar compared to the untouched upper left first molar. Statistically, only the sham and NGF groups were significantly different. Error bars represent the standard error of the mean difference. *P < 0.05 and **P < 0.01.

The linear measurement of the maximum mesial root length reveals significant root shortening of the experimental right first molar compared to the untouched left first molar for all experimental groups (Figure 6). Mesial root length reduction was significantly different between the sham and NGF groups only.

**Figure 7:**
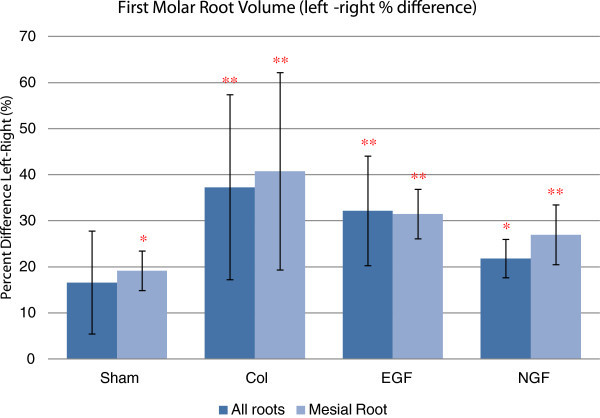
**Comparison of the left and right first molar root volume.** Estimated mean percentage difference between the right and left first upper molar for total root volume and mesial root volume. All experimental groups showed a significant reduction in root volume (total root volume and mesial root volume) compared to the untouched left molar. Utilisation of a collagen membrane, with or without the incorporation of a growth factor, had a tendency to reduce root volume, although this was not statistically significant. Error bars represent the standard error of the mean difference. *P < 0.05 and **P < 0.01.

*Volumetric measurement* of the total root structure and the mesial root separately reveals a significant reduction in the root volume of the replanted upper right first molar compared to the untouched upper left first molar (Figure 7). Additionally, there were no significant between-group differences in root volume, although there was a tendency for experimental groups utilising a collagen membrane (collagen, EGF and NGF) to have reduced root volume compared to the sham group.

**Figure 8:**
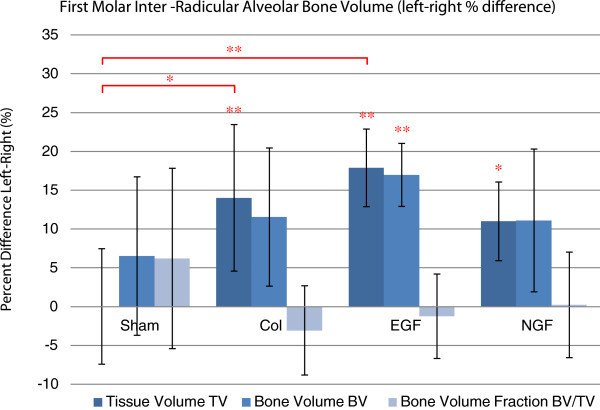
**Comparison of the left and right first molar inter-radicular alveolar bone volume.** Estimated mean percentage difference of inter-radicular alveolar bone volume and bone volume fraction between the right and left upper first molars. There was a significant reduction in the tissue volume for the groups collagen (Col), EGF and NGF, while only EGF had a significant reduction in bone volume. Bone volume fractions were not altered significantly in any experimental group. Between-group assessment revealed a significant difference in the tissue volume between the sham group and the collagen and EGF groups. Error bars represent the standard error of the mean difference. *P < 0.05 and **P < 0.01.

Volumetric assessment reveals a reduction in the tissue and bone volume in the inter-radicular region of the upper right first molar compared to the untouched upper left first molar (Figure 8). This reduction is greatest for the experimental groups utilising a collagen membrane (Collagen, EGF and NGF) with a statistically significant reduction in tissue volume for Collagen, EGF and NGF groups while only the EGF group had a significant reduction in bone volume.

Between-group comparison that reveals tissue volume in the inter-radicular region of the upper right first molar was significantly reduced in the collagen and EGF groups compared to the sham group but not so for the NGF group. There was no statistically significant difference in the bone tissue fraction for any of the four experimental groups indicating that bone density did not alter significantly (Figure 8).

### Histological analysis

**Figure 9:**
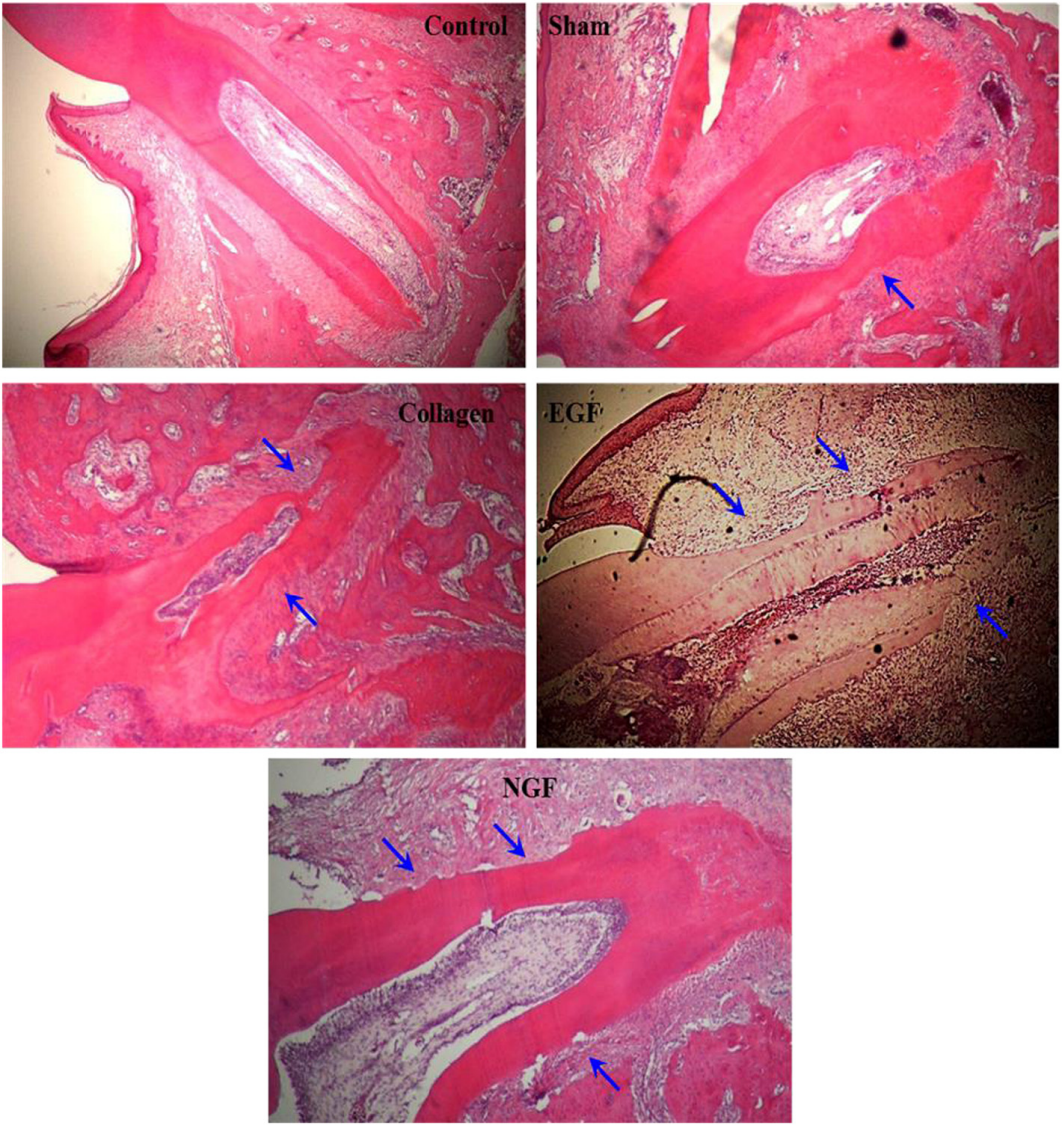
**Representative photomicrographs demonstrating root resorption of the mesial root of the maxillary first molars.** Photomicrographs (×40 H & E) of the mesial root of the upper right first molar from the experimental groups (sham, collagen, EGF and NGF) and a representative photomicrograph of the upper left first molar mesial root (control). No root resorption was noted in the control while all experimental groups showed resorption defects of varying extent (blue arrows). Resorption was particularly severe in the experimental teeth involving a collagen membrane (collagen, EGF and NGF). Ankylosis was not detected in the histology sections assessed from any group.

Histological assessment of the root morphology of the untouched left molar (control) consists of a thin layer of acellular cementum covering the root surface without discontinuity, a functionally orientated PDL and no evidence of root resorption or ankylosis (Figure 9). The mesial root of the sham group is covered mainly by cementum and a functionally orientated PDL, although isolated regions of root resorption were present. Replanted molars involving the use of a collagen membrane (collagen, EGF and NGF groups) had extensive root resorption, involving over half of the root surface, with minimal cementum coverage. Ankylosis could not be detected in any of the histological sections assessed.

**Figure 10:**
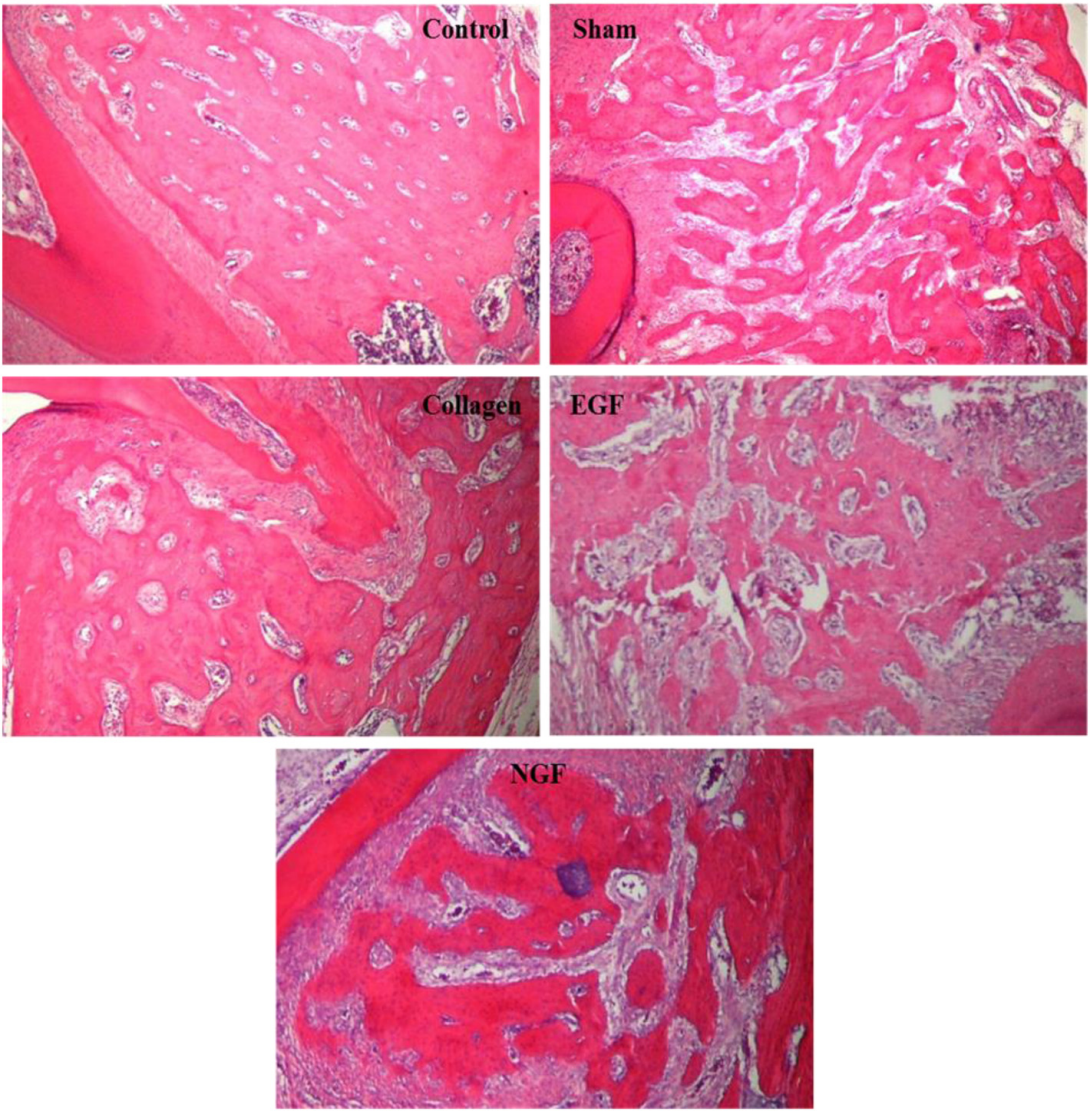
**Representative photomicrographs demonstrating inter-radicular alveolar bone architecture of the maxillary first molars.** Photomicrographs (×40 H & E) of the inter-radicular alveolar bone of the upper right first molar from the experimental groups (sham, collagen, EGF and NGF) and a representative photomicrograph of the upper left first molar inter-radicular alveolar bone (control). The control photomicrograph shows the normal architecture of the alveolar process with thick bone trabecula and small medullary spaces. Photomicrographs of the experimental groups (sham, collagen, EGF and NGF) show a fragmented alveolar process consisting of thin bone trabecular and wide medullary spaces.

The inter-radicular alveolar bone of the untouched control has a histological appearance of well-organised thick bone trabecula interspersed with small medullary spaces (Figure 10). The histological appearance of the alveolar bone in the replantation groups (sham, collagen, EGF and NGF) is more fragmented with thin trabecular bone and large medullary spaces (Figure 10). Additionally, the transplanted teeth were surrounded by the granulation tissue containing macrophages, fibroblastic cells, many newly formed blood vessels and sparse and thin connective tissue fibres.

**Figure 11:**
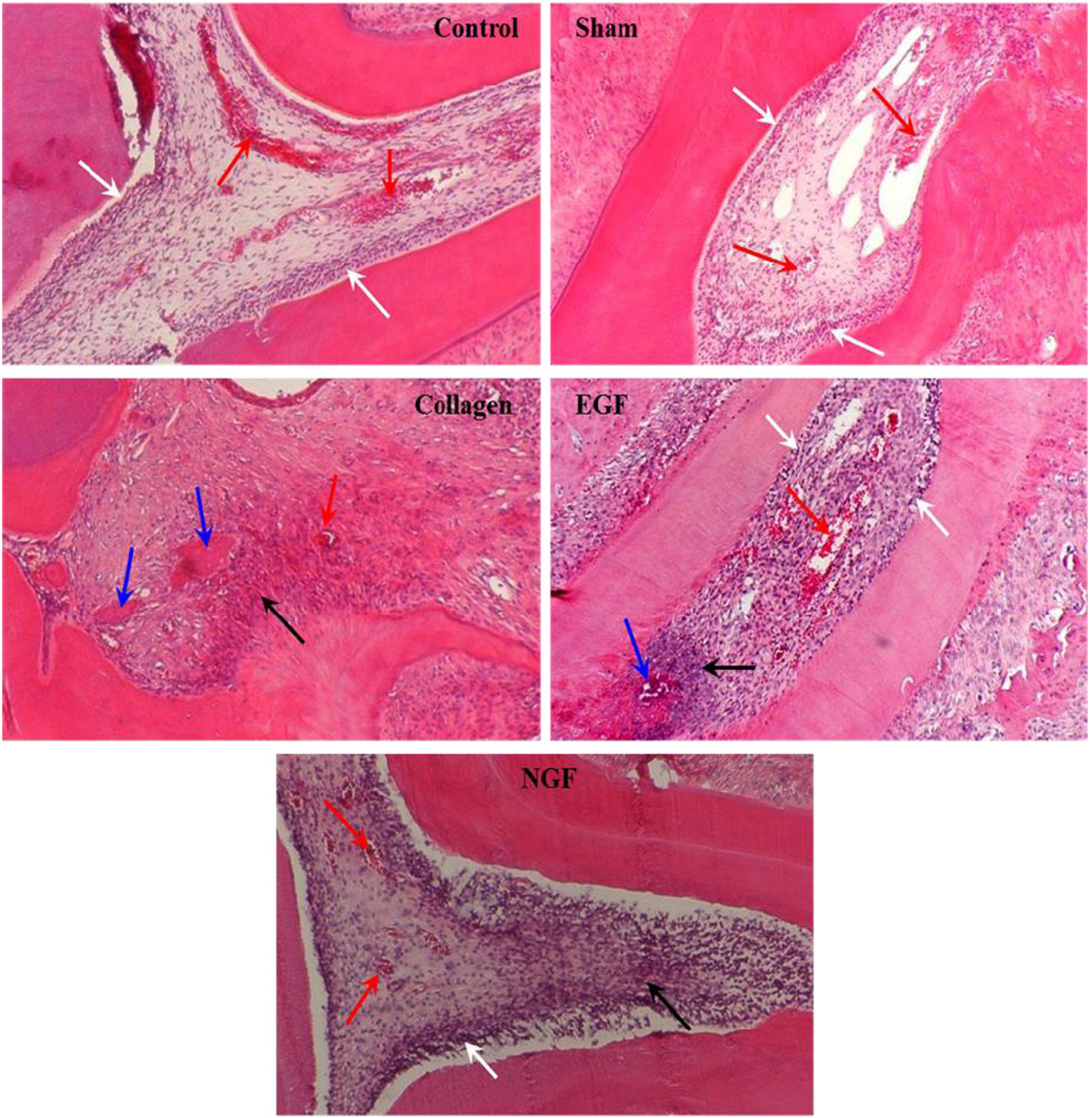
**Representative photomicrographs demonstrating pulpal appearance of the mesial root of the maxillary first molars.** Photomicrographs (×100 H & E) of the pulp chamber from the mesial root of the upper left first molar (control) and upper right first molar of the experimental groups (sham, collagen, EGF and NGF). Note the normal appearance of the control with a well-vascularised pulp (red arrows) and well-defined odontoblastic layer (white arrows). Vascularisation in the experimental groups is reduced compared to the control, especially for the collagen and NGF groups, with the EGF group showing improved vascularisation compared to other groups involving a collagen membrane. An odontoblastic layer is present in the sham, EGF and NGF groups, although these are not at as well defined as the control group. The pulp of the collagen group had lost this odontoblastic layer and was found to contain extensive inflammatory regions (black arrow) and even some hard tissue formation (blue arrows). EGF and NGF groups also had increased inflammatory cell infiltrate (black arrows), while the EGF group had signs of hard tissue formation (blue arrow). However, the pulpal architecture and cell organisation in the NGF group more closely resembled that of the control group compared to the other experimental groups involving a collagen membrane (collagen and EGF groups).

The histological appearance of the pulp from the control molars consisted of an even distribution of cells, a well-defined odontoblastic layer, no inflammatory cells, and a high density of blood vessels (Figure 11). The pulp of the molars in the sham group closely resembled that of the control molars with an odontoblastic layer lining the pulp wall, although not as well defined as the control molars, and minimal inflammatory cells were present. Pulp from the molars in the collagen, EGF and NGF groups showed a general loss of pulpal organisation with no to minimal odontoblastic layer present, extensive amount of inflammatory cells and isolated regions of hard tissue formation (Figure 11). However, the histological organisation of the pulpal cells in the NGF group more closely resembled that of the control group compared to the other experimental groups involving a collagen membrane. Revascularisation was observed in all the replanted molars, as shown by the presence of red blood cells and blood vessels within the pulp chamber. However, vascularisation was reduced in all replanted molars compared to the control molars. This was significantly evident in replanted molars in the collagen and NGF groups while the vascularisation in the EGF group appeared to be greater although not to the level of the sham group (Figure 11).

## Discussion

### Periodontal and root healing

It was hoped that the incorporation of EGF and NGF into the alveolar socket prior to molar replantation in the rat model would aid in periodontal healing. However, no benefit of incorporating these growth factors was observed in this study. Replanted molars in the EGF and NGF groups had similar rates of extensive root resorption as did the collagen only group.

Micro-CT and histological assessment revealed that all replanted rat molars had significantly reduced root lengths and root volumes compared to the untouched left molar. This suggests that the addition of the collagen membrane, with or without EGF and NGF, to the alveolar socket prior to replantation negatively affected root healing and development. It is known that root growth is dependent upon the coordinated activity of Hertwig's epithelial root sheath, the pulp and the PDL cells [33]. Continued root development after transplantation can only be expected if Hertwig's epithelial root sheath is preserved around the apices suggesting that trauma was sustained to the root sheath during transplantation, especially in the experimental groups involving a collagen membrane.

### Bone healing

Although all the molar replantations were carried out as atraumatic as possible, some damage to the dentoalveolus inevitably occurred. This was reflected by a general tendency for increased alveolar trabeculisation and fragmentation, and decreased bone and tissue volumes in all the experimental groups. The incorporation of EGF or NGF, when compared to the collagen only group, showed no significant effect on alveolar healing. Additionally, compared to the sham group, the use of a collagen membrane with or without any growth factors seemed to make the dentoalveolar healing worse. It appears that the presence of the relatively rigid collagen membrane may have interfered with the normal alveolar bone healing, and any possible benefit the growth factors may have had. This suggests that the use of collagen membrane as a protein carrier may not be ideal for *in vivo* studies involving the rat model.

A variety of new injectable materials such as hydrogels are being developed for growth factor delivery applications. These injectable gels are especially attractive because they can allow for minimally invasive delivery of inductive molecules which is beneficial when dealing with delicate structures such as rat alveolar bone and epithelial root sheaths [34]. However, King et al. [35] reported that the more slowly dissolving collagen membrane carrier system allowed for more prolonged exposure to BMP-2 while being able to maintain the growth factor within the required region better than a gel carrier system when assessing wound healing of periodontal fenestration defects in a rat model. Further research is therefore required into the gel carrier systems before they are suitable for use in a clinical setting.

### Pulp healing

An important factor affecting the survival rate of transplanted teeth is the response of the pulp to the trauma sustained. If pulpal necrosis occurs, there is the possibility of periapical inflammation and inflammatory root resorption, leading to the eventual loss of the transplant [36,37]. Immature roots consisting of a wide, open apical foramen have improved rates of healing compared to mature teeth [38,39]. Since the root development of the maxillary first molars from four-week old rats is immaturely developed, the prognosis of pulpal healing should be good. This was observed in the current study with all the replanted molars demonstrating histologically successful pulpal revascularization.

It was interesting to observe that the pulps from the EGF group showed improved vascularisation compared to the collagen only and NGF groups. EGF is known to promote angiogenesis *in vivo*[40] and EGF receptors have been localised in the dental pulp in the rat [41]. Derringer and Linden have shown that the addition of anti-h EGF to pulp cell culture reduced the angiogenic response with significantly fewer micro-vessel formations [42]. Therefore, we hypothesise that the addition of EGF may have accelerated pulpal revascularisation after dental replantation and induced earlier pulpal healing. Further investigation will be required over multiple time points to assess the actual vascularisation rate and to determine if this enhanced pulpal revascularization will also occur in mature teeth with smaller apical foramina.

Of equal importance was the observation that the pulpal architecture and cell organisation in the NGF group which more closely resembled that of the control group compared to the collagen only and EGF groups. *In vitro* studies demonstrate that nerve fibres selectively grow only in a local environment containing NGF and show preferential orientation following NGF concentration gradients [24]. The developmental role for NGF is consistent with the early presence of NGF receptor (NGF-R) in the pulp. Upon binding to NGF-R, NGF could exert a wide range of effects on odontogenic cells by providing the positionally and temporally correct microenvironment. This study appears to support the idea that in addition to functions concerning dental neurobiology, NGF may influence the timing, sequence and position for numerous dental cell phenotypes localised in the healing dental pulp.

## Conclusions

Possible beneficial effects of incorporating growth factors into the socket of a replanted molar in the rat model include improved pulpal vascularisation with the use of EGF and improved pulp cell organisation with NGF. No beneficial effects were observed in regards to root, alveolar or periodontal healing. In addition, the use of a collagen membrane carrier appeared to negatively affect the healing of the replanted molar.

## Competing interests

The authors declare that they have no competing interests.

## Authors’ contributions

FF contributed to the design of the study, animal handling, and data acquisition. Data interpretation, and drafted the manuscript. ESMA contributed to the design of the study, animal handling, and data acquisition. RRL participated in the design of the study, animal handling and data acquisition. KM participated in the design of the study and performed the statistical analysis. MG conceived the study, participated in its design and participated in manuscript formatting. All authors read and approved the final manuscript.
